# Systematic classification of non-coding RNAs by epigenomic similarity

**DOI:** 10.1186/1471-2105-14-S14-S2

**Published:** 2013-10-09

**Authors:** Mikhail G Dozmorov, Cory B Giles, Kristi A Koelsch, Jonathan D Wren

**Affiliations:** 1 Oklahoma Medical Research Foundation, Oklahoma City, Arthritis and Clinical Immunology Research Program, 825 N.E. St, Oklahoma City, OK 73104-5005, USA; 2 University of Oklahoma Health Sciences Center, Department of Biochemistry and Molecular Biology, 940 Stanton L. Young Blvd, OK 73104-5005, USA

**Keywords:** ncRNA, non-coding RNA, epigenetics, genome, ENCODE, GenomeRunner

## Abstract

**Background:**

Even though only 1.5% of the human genome is translated into proteins, recent reports indicate that most of it is transcribed into non-coding RNAs (ncRNAs), which are becoming the subject of increased scientific interest. We hypothesized that examining how different classes of ncRNAs co-localized with annotated epigenomic elements could help understand the functions, regulatory mechanisms, and relationships among ncRNA families.

**Results:**

We examined 15 different ncRNA classes for statistically significant genomic co-localizations with cell type-specific chromatin segmentation states, transcription factor binding sites (TFBSs), and histone modification marks using GenomeRunner (http://www.genomerunner.org). P-values were obtained using a Chi-square test and corrected for multiple testing using the Benjamini-Hochberg procedure. We clustered and visualized the ncRNA classes by the strength of their statistical enrichments and depletions.

We found piwi-interacting RNAs (piRNAs) to be depleted in regions containing activating histone modification marks, such as H3K4 mono-, di- and trimethylation, H3K27 acetylation, as well as certain TFBSs. piRNAs were further depleted in active promoters, weak transcription, and transcription elongation regions, and enriched in repressed and heterochromatic regions. Conversely, transfer RNAs (tRNAs) were depleted in heterochromatin regions and strongly enriched in regions containing activating H3K4 di- and trimethylation marks, H2az histone variant, and a variety of TFBSs. Interestingly, regions containing CTCF insulator protein binding sites were associated with tRNAs. tRNAs were also enriched in the active, weak and poised promoters and, surprisingly, in regions with repetitive/copy number variations.

**Conclusions:**

Searching for statistically significant associations between ncRNA classes and epigenomic elements permits detection of potential functional and/or regulatory relationships among ncRNA classes, and suggests cell type-specific biological roles of ncRNAs.

## Background

The advent of novel high-throughput technologies such as next-generation sequencing (NGS) has empowered the gathering of transcriptional data of unprecedented quantity and quality. It has lead to the discovery that ~75% of the human genome is transcribed to RNA at some point in certain cell types [1,2], with other estimates being as high as 90% [3]. Only ~1-2% of these transcripts are translated into protein form [4,5], while the remainder are classified as non-coding RNAs (ncRNAs) [6,7]. The fact that ncRNAs are relatively abundant, expressed in a developmentally regulated fashion [8,9], and often exhibit precise sub-cellular localization [2] supports the notion that they play important biological roles. In particular, their ability to base-pair with other transcripts and regions suggests they may be responsible for a variety of regulatory functions [10].

Attempts to untangle the complex landscape of ncRNAs have led to crude classification of ncRNAs based on their length (small, 18-31nt; medium, 31-200nt; and long, >200nt) [11], function (housekeeping ncRNAs such as ribosomal (rRNAs), transfer RNAs (tRNAs)), regulatory potential (microRNAs (miRNAs), long non-coding RNAs (lncRNAs)) [12], and subcellular localization (small nuclear RNAs (snRNAs), small nucleolar RNAs (snoRNAs), cytoplasm-located piwi-interacting RNAs (piRNAs), and short interfering RNAs (siRNAs)). Other unusual ncRNA species such as trans-spliced transcripts, macroRNAs that encompass enormous genomic distances, and multi-gene transcripts that encompass several genes or even the whole chromosome further confound efforts for systematic classification [13-15]. In reality, however, clear categorization of ncRNA classes has been quite difficult, as many ncRNA transcripts often share the properties of multiple categories.

ncRNAs can also be broadly categorized by genomic properties such as 1) sense or antisense transcripts, when the ncRNA transcript co-localizes with exons of another transcript on the same or the opposite strand, respectively, 2) bidirectional, when the expression of the ncRNA and of a neighboring coding transcript in the opposite strand are initiated in close proximity, 3) intronic, when the ncRNA arises from an intron of another transcript, and 4) intergenic, when the ncRNA is localized between two coding transcripts [10,12,16]. Other ncRNA classes, such as promoter-associated RNAs (PARs) and enhancer RNAs (eRNAs) are also being characterized [17,18]. These considerations alone suggest the role of genomic organization in the biogenesis of ncRNAs is more complex than previously thought [2].

Recent years have seen a rapid growth of publicly available data on genome organization and functional annotation. The Encyclopedia of DNA Elements (ENCODE) project has been actively cataloging functional elements in the human genome, such as cell type-specific histone modification profiles, chromatin states, and transcription factor binding sites [3]. In this study we will refer to these functional and regulatory regions as epigenomic elements, i.e., genomic data other than nucleotide sequence that describes functions, properties, or experimental values associated with genome regions [19-23]. Although the precise definition of the word "epigenomics" is hotly debated [24] and is often narrower in scope, we feel our broader definition of epigenomic elements is suitable to convey the concepts of our research to the readers. The aim of the current study was to use the ENCODE data, such as cell type-specific histone modification profiles, chromatin states, and experimentally validated transcription factor binding sites to examine how epigenomic elements statistically significantly associated with different classes of ncRNAs may reveal functional roles and relationships among ncRNA classes.

We investigated whether the genomic coordinates of 15 ncRNA classes significantly overlapped with or were depleted in a total of 420 annotated epigenomic elements. We found that piRNA and tRNA ncRNAs were strongly under- and over-represented with the majority of epigenomic elements examined, respectively. Other ncRNA classes showed less extreme enrichments, but we were able to confirm known and identify new statistically significant ncRNA class-specific epigenomic associations. Clustering ncRNA classes by the significance of their epigenomic associations captures their known hierarchy, providing a means to utilize epigenomic background for classification purposes. In summary, our study demonstrates a means to use genome annotation data to identify regulatory commonalities and differences among genomic and epigenomic elements.

## Methods

### ncRNA data

**Figure 1:**
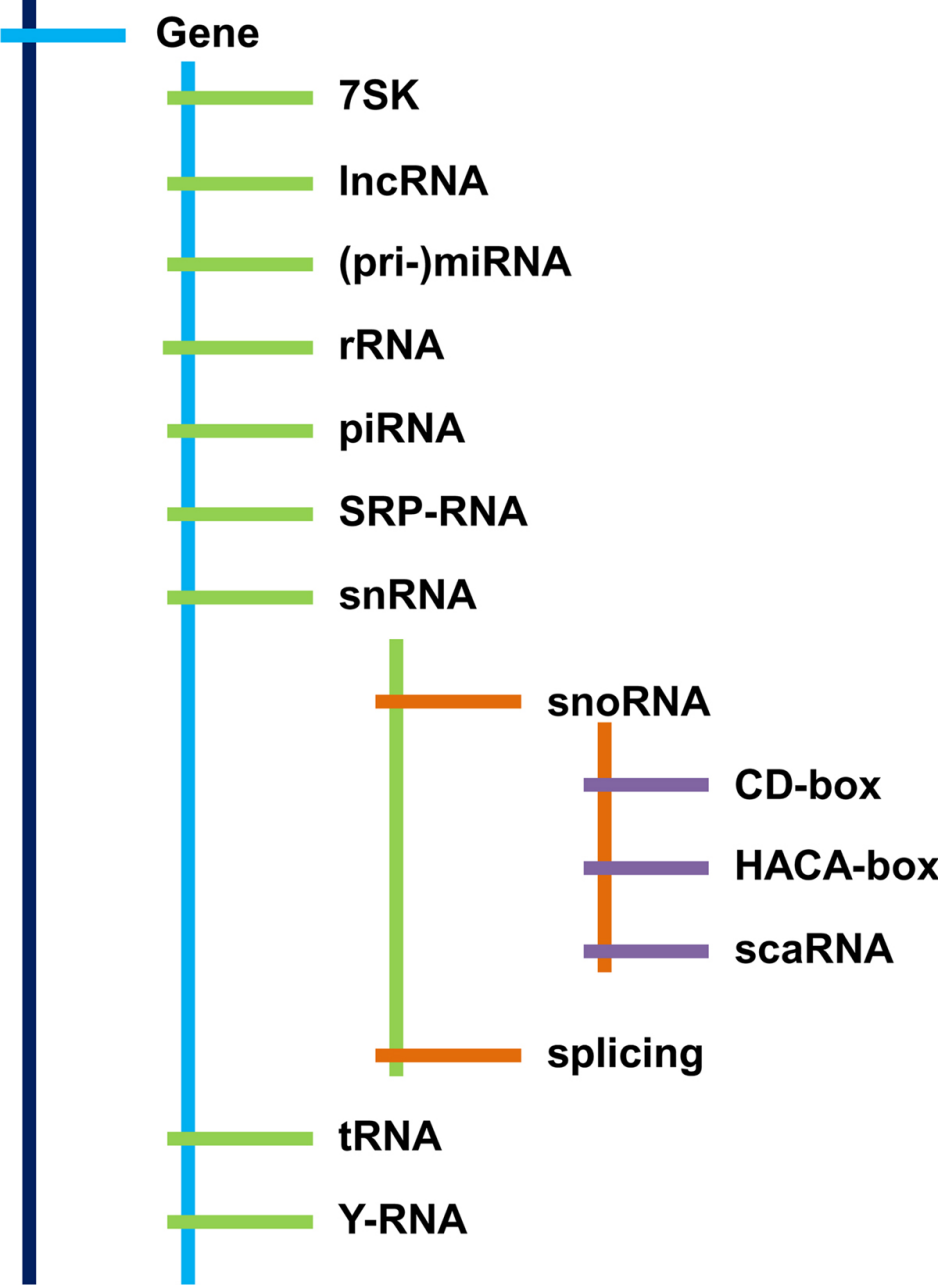
**The hierarchy of ncRNA classes**. Adapted from [45].

GRCh37/hg19 genome assembly coordinates for different classes of non-coding RNAs were extracted from miRbase (miRNAs and pri-miRNAs), UCSC genome database (piRNAs), and RFAM (all other classes) using custom scripts available at git://wrenlab.org/ncRNA-loci. piRNAs were extracted from the UCSC knownGenes table by string matching on "*piRNA*", and RFAM categories were used for RFAM-derived elements. We ignored ncRNA classes with less than 50 members, as their analysis did not show any statistically significant associations (data not shown). The relationship among ncRNA classes is shown on Figure 1. A total of 16,701 ncRNAs were analyzed (Table 1).

**Table 1:** The ncRNA classes used in the current study

ncRNA abbreviation	Parent family	Source	Description	Function	Counts	Min length	Mean length	Max length
7SK	Gene	RFAM	7SK RNA	Part of snRNP complex; involved in control of transcription elongation.	316	61	293	374
C/D box	snoRNA	RFAM	CD-box RNA	Localized to nucleolus, involved in methylation of rRNA.	509	30	99	238
H/ACA box	snoRNA	RFAM	HACA-box	Localized to nucleolus, involved in pseudouridylation of rRNA.	440	52	130	329
lncRNA	Gene	RFAM	Long non-coding RNA	A non protein-coding transcript with > 200 nt.	217	36	144	463
miRNA	Gene	miRbase	micro RNA	A short, hairpin-shaped RNA that usually suppresses translation of target mRNA by binding to 3' UTR.	2233	15	22	27
piRNA	Gene	UCSC	piwi-interacting RNA	A large class of 26-31 nt RNA suggested being involved in transposon silencing.	2152	20	47	8302
pri-miRNA	Gene	miRbase	primary microRNA	A transcript containing one or more microRNAs before processing by Drosha complex.	1595	41	83	180
rRNA	Gene	RFAM	ribosomal RNA	RNA which is directly incorporated into the ribosome (distinct from mRNA encoding ribosomal proteins).	611	34	119	1860
scaRNA	snoRNA	RFAM	small Cajal body-specific RNA	Located in the nucleolus, and involved in methylation and pseudouridylation of spliceosomal RNAs.	52	82	153	419
snoRNA	snRNA	RFAM	small nucleolar RNA	RNA located in the nucleolus, mostly involved in modification of other RNAs, such as rRNA or spliceosomal RNA.	1001	30	115	419
snRNA	Gene	RFAM	small nuclear RNA	Small RNA located in the nucleus, involved in the spliceosome, RNA modification, or other functions.	3024	30	109	419
spliceosomal	snRNA	RFAM	spliceosomal RNA	RNA forming part of the spliceosome complex.	1812	49	111	229
SRP-RNA	Gene	RFAM	signal recognition particle RNA	RNA forming part of the SRP complex. The SRP complex targets proteins to their proper subcellular localization by recognizing the signal recognition peptide tag on proteins.	941	201	286	361
tRNA	Gene	RFAM	transfer RNA	Transfer amino acids to the ribosome for protein construction.	905	59	72	107
Y-RNA	Gene	RFAM	Y RNA	Components of the Ro RNP complex; may repress Ro activity. As an independent function, is also required for DNA replication.	893	57	102	148
Total					16701			

### Functional/Regulatory elements data

As part of our interest in automating the search for biologically meaningful correlations among high-throughput and high-information data [25-27], we developed GenomeRunner (http://sourceforge.net/projects/genomerunner), a software program that searches for statistically significant co-localization between a set of genomic regions of interest (ncRNA classes in this paper) and sets of annotated genomic features (epigenomic elements, e.g., genomic regions in the GM12878 cell line where H3K4me1 histone modification sites were found) [28]. We tested each different class of ncRNAs to see if they were statistically significantly associated with or depleted in three groups of epigenomic elements: Chromatin State Segmentation by HMM from ENCODE/Broad, Histone Modifications by ChIP-seq from ENCODE/Broad Institute, and Experimentally validated Transcription Factor ChIP-seq from ENCODE (Additional files 1, 2). A total of 420 epigenomic elements were obtained from the UCSC genome database [29], and stored in a local MySQL database accessible for the GenomeRunner community.

### Enrichment analysis

Briefly, sets of ncRNAs were tested for genomic overlap of at least 1 nucleotide with 420 epigenomic elements. We first calculated the total number of members from an ncRNA class that overlapped with an epigenomic element. If a given ncRNA overlaps with >1 epigenomic element of the same type, it is only counted once, to reflect the fact of overlap. In other words, overlap counting is ncRNA-centric, e.g., if a tRNA overlaps with 3 NFKB binding sites, only one is added to the tRNA-NFKB overlap counter.

We then performed random sampling from the total pool of all 15 classes of ncRNAs, selecting the same number of random ncRNAs as in the class being analyzed. The use of all ncRNAs as a background enables us to search, specifically, for associations that might distinguish one ncRNA class from the rest. This background selection also restricts random sampling to avoid low complexity genomic regions, such as transposable elements, duplications and inversions, repeats, comprising up to 47% of the human genome [30]. We selected a random number of ncRNA elements according to the size of each ncRNA class and performed 1000 such random samplings, estimating the average number and variance of random overlapping. A Chi-square test was used to determine whether there was a significant difference between the total number of co-localization for the ncRNA class as compared with what could be expected by random chance.

### Transformation of p-values

To emphasize epigenomic elements most significantly associated with an ncRNA class, p-values were adjusted for multiple testing using the Benjamini-Hochberg procedure [31]. For easier visualization and comparison, we converted p-values into decimal scale by -log10-transformation. A "-"sign was added if a p-value represents an underrepresented association. This allows representation of significant associations in an intuitive format - larger numbers equal more statistically significant overrepresented associations of a ncRNA class with an epigenomic element, while smaller negative numbers represent more statistically significant under-represented associations.

### Clustering and visualization

The transformed p-values can be directly visualized with a blue-yellow gradient representing under- and over-represented associations, respectively. A n × m matrix of transformed p-values, where n is the number of ncRNA classes and m is the number of epigenomic elements, was assembled (Additional file 3). Epigenomic elements showing no statistically significant associations (p-value cutoff 0.01, unless otherwise specified) with at least one ncRNA class were removed. Further, epigenomic elements showing consistent associations across the ncRNA classes (standard deviation of the transformed p-value distribution is less than 2) were also filtered. These filtering steps simplify visualization and allow a reader to focus on the most significant epigenomic associations differentially enriched among ncRNA classes.

Hierarchical clustering was performed using "maximum" distance to measure dissimilarity between rows and columns, and the "ward" agglomeration method [32]. Clustering and visualization were performed within R computing environment [33].

### Pair-wise correlation analysis

We compared epigenomic similarities and differences among ncRNA classes. That is, pair-wise correlations between all ncRNA class-specific transformed p-values were measured using Pearson's correlation coefficient. We expect ncRNA classes showing similar enrichment patterns with epigenomic elements to correlate with positive Pearson's correlation coefficient close to 1, while ncRNA classes differentially associated with epigenomic elements would show negative Pearson's correlation coefficient. Combining these Pearson's correlation coefficients into n × n matrix, where n is the number of ncRNA classes, allows clustering and visualization of epigenomic similarities among ncRNA classes as describe above, using blue/yellow gradient to highlight negative/positive correlations, respectively.

## Results

**Figure 2:**
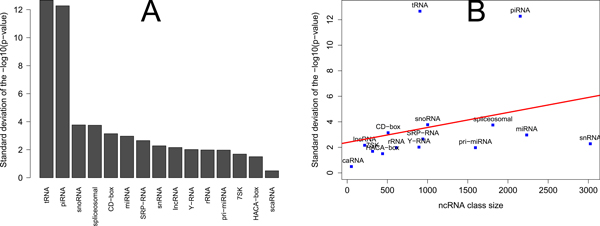
**Variability of enriched associations per ncRNA class**. A) The standard deviation of ncRNA classes enrichment p-values, adjusted for multiple testing, tested for associations with all epigenomic elements. B) Positive correlation between the size of an ncRNA class and the variability of their associations with all epigenomic elements.

The number of members per ncRNA class varies considerably, ranging from 52 scaRNAs to 3,024 small nuclear RNAs (Figure 1, Table 1). This variability in class size was accompanied by variability in their statistically significant enrichments versus all 420 epigenomic features. The tRNA and piRNA classes showed the largest variability of the enrichment/depletion p-values, after correction for multiple testing (Figure 2A).

There was a positive correlation between the size of ncRNA classes and the standard deviation of the p-value (Pearson's correlation coefficient = 0.25), although it was not significant (*p *= 0.487), likely due to the extreme p-value variability in tRNA and piRNA classes. Removing those classes increased Pearson's correlation coefficient to 0.58 (*p *= 0.134). Notable, that both classes did not show extreme size or length abnormalities, being comparable in size with snoRNA and miRNA classes (Table 1, Figure 2B), suggesting strong epigenomic associations driving p-value variability.

### Pair-wise correlation analysis of epigenomic associations reveal known and novel relationships among ncRNA classes

**Figure 3:**
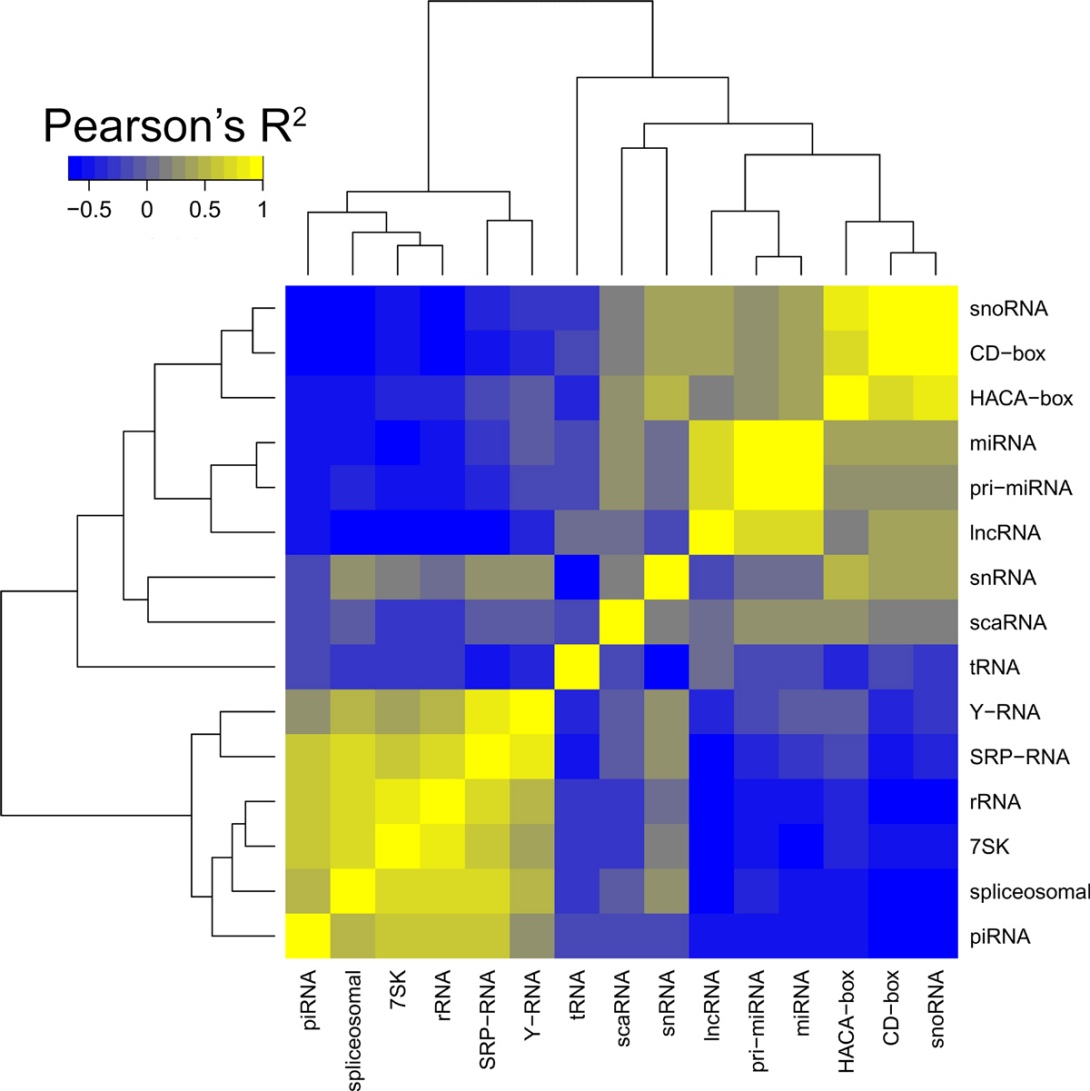
**Correlations among the ncRNA classes based on their enriched or depleted associations with all epigenomic elements**. If two classes exhibit similar enrichment/depletion patterns, they would be positively correlated (yellow gradient). Patterns of the opposite associations will be negatively correlated (blue gradient).

We investigated how similar ncRNA classes were across all their associations with epigenomic elements. That is, p-values for each ncRNA-epigenomic element were calculated and their similarities across all 420 elements analyzed were quantified by Pearson's correlation coefficient (Figure 3). Out of 420 elements analyzed, 64 did not show statistically significant associations with any of the ncRNA classes. Therefore, 356 were used for the global correlation analysis (Additional file 4).

Expectedly, snoRNA, C/D-box and H/ACA-box ncRNA classes shared similar epigenomic associations, being subgroups of a larger snRNA class (Figure 1). Yet scaRNAs, a subgroup of snoRNAs, did not share similar significant associations. This may be, in part, due to size differences in the groups (see Table 1). Along with tRNA class, scaRNAs had epigenomic associations distinct from other ncRNA classes. Spliceosomal, rRNA, 7SK, piRNA, SRP-RNA and Y-RNA classes formed the largest group with similar epigenomic associations.

While ncRNA classes that cluster together by their epigenomic associations provide a good means to assess potential functional/regulatory similarities, each class can also have anti-correlated epigenomic associations. Pairs of ncRNA classes best (anti) correlating with each other are listed in Table 2.

**Table 2:** Pearson's correlation coefficient for pairs of ncRNA classes best correlated and anticorrelated by their epigenomic associations

ncRNA class	best correlates with	at Pearson's correlation coefficient	and best anticorrelates with	at Pearson's correlation coefficient
7SK	rRNA	0.71	miRNA	-0.66
C/D box	snoRNA	0.94	Spliceosomal	-0.65
H/ACA box	snoRNA	0.65	piRNA	-0.24
SRP-RNA	spliceosomal	0.69	lncRNA	-0.55
Y-RNA	SRP.RNA	0.60	CD-box	-0.32
lncRNA	miRNA	0.54	Spliceosomal	-0.58
miRNA	pri.miRNA	0.91	7SK	-0.66
piRNA	rRNA	0.61	CD-box	-0.62
pri-miRNA	miRNA	0.91	7SK	-0.54
rRNA	spliceosomal	0.75	CD-box	-0.61
scaRNA	miRNA	0.04	7SK	-0.23
snRNA	snoRNA	0.26	tRNA	-0.54
snoRNA	CD.box	0.94	piRNA	-0.58
spliceosomal	rRNA	0.75	CD-box	-0.65
tRNA	lncRNA	0.08	snRNA	-0.54

### piRNAs are depleted, while tRNAs are enriched in histone modification marks

**Figure 4:**
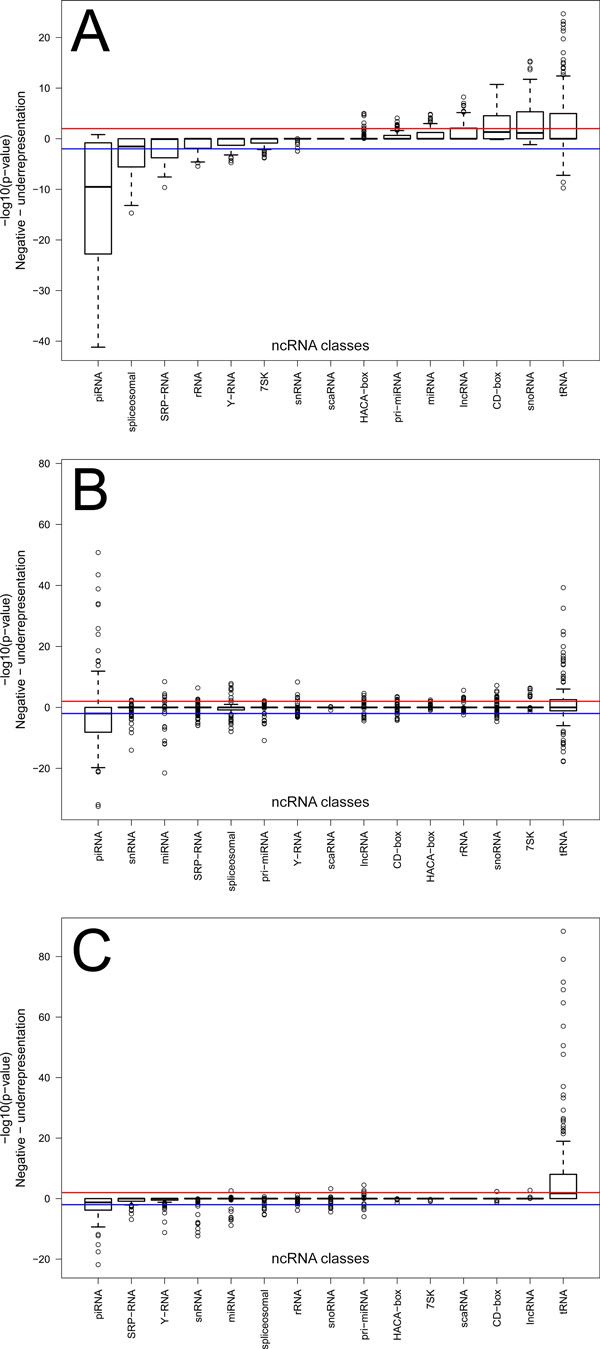
**Boxplots of -log_10_(p-value) distributions for different classes of ncRNAs**. Red/blue horizontal lines represent p-value = 0.01 threshold for over- and underrepresentation, respectively. P-values were multiple testing corrected using Benjamini-Hochberg procedure. A) Histone modification marks associations; B) Chromatin states associations; C) Transcription factor binding sites associations.

Combinations of histone modifications mark the boundaries of transcriptionally active regions, and are associated with distinct regulatory sites [34]. We expected to observe both enriched and depleted associations of ncRNA classes with histone marks. Surprisingly, ncRNA classes showed either preferentially enriched or depleted statistically significant associations with histone modification marks. piRNAs, spliceosomal, SRP-RNAs, rRNAs, Y-RNAs were predominantly depleted in histone modification marks from the 14 cell lines examined. tRNAs, H/ACA-box, pri-miRNAs, miRNAs, lncRNAs, C/D-box and snoRNAs were enriched in histone modification marks. Notably, piRNAs and tRNAs showed the largest variability in enrichment (Figure 4A).

**Figure 5:**
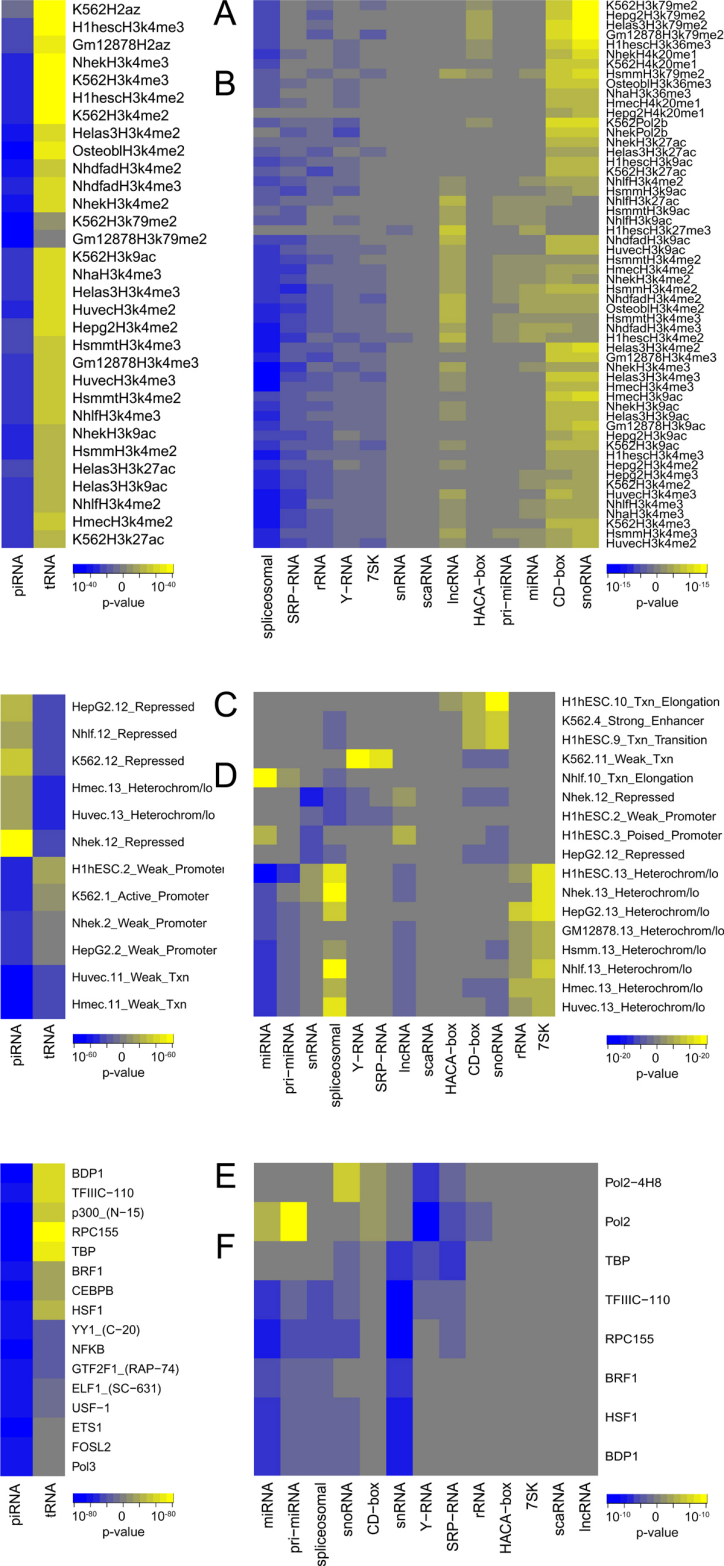
**Heatmaps of -log_10_(p-value) enriched associations**. Blue-yellow gradient highlights the significance of under-/overrepresented associations of ncRNA classes (X axis) with corresponding epigenomic elements (Y axis). Panels A), C), and E) represent associations of piRNA and tRNA classes with the Histone modification marks, chromatin state segmentation, and transcription factor binding sites, respectively. Only associations with p.adj<10^-20 ^are shown for clarity. Panels B), D), and F) show the same associations for other ncRNA classes

Transfer RNAs and piwi-interacting RNAs showed the strongest anti-correlated associations with epigenomic elements (Figure 5A, Additional files 4, 5). The tRNAs were strongly enriched in the actively transcribed genomic regions, permissive and activation-related H3K4 di- and trimethylation, H3K9 and H3K27 acetylation marks, and H2az histone variant. Contrary to the overall picture of active transcription regions associated with tRNAs, they were also associated with the transcription insulator CTCF binding sites (Additional file 3) that blocks communication between promoters and downstream genes [35]. Although we identified that tRNAs tend to be enriched in regions with CTCF insulators, whether or not CTCF binds in these regions remains to be determined.

Conversely, piRNAs were associated with heterochromatin regions in the genome and underrepresented in active promoter regions (Figure 5A, Additional files 4, 5). piRNAs were depleted in the same types of histone marks the tRNAs were enriched in. That is, piRNAs were depleted in activation-related H3K4 mono-, di- and trimethylation marks, H3K9ac, H3k27ac and H3k36me3 marks, and the H2az histone variant. Yet, the association of piRNAs with transcriptionally silent regions of the genome is blurred by their depletion in H4K20me1, a marker of silent chromatin, and in H3k27me3 marker of gene repression.

Heatmap visualization helps summarize over- and underrepresentation trends for other ncRNA classes (Figure 5B, Additional files 4, 6). The H3K4 di- and trimethylation marks were the most frequently differentially enriched, followed by H3K9ac. We did not observe bias in the cell type-specific origin of these histone marks, although K562, NHEK, H1hESC, Hela-S3 and HepG2 specific marks dominated the heatmap. The driving force behind the clustering appeared to be the histone marks themselves. This can be seen from groups of H3K79me2, H3K4me2 and H3K4me3 showing similar enrichment patterns, thus clustered together, on the heatmap.

Interestingly, even though C/D box and H/ACA box RNAs are subclasses of snoRNAs, the H/ACA box subclass was not enriched for most of the associations that either snoRNAs or C/D box RNAs were, in general. This is in contrast to the general similarity of associated features shown in Figure 5B and indicates that one of the factors that may separate the two snoRNA subclasses is their regulatory locations. The only mark H/ACA box and C/D boxes seemed to both be enriched in was H3K79me2 which has been associated with the cell cycle. This suggests that H/ACA and C/D box RNAs may be orchestrated similarly by histone modification marks during the cell cycle, but not beyond.

### piRNAs are enriched in the repressed chromatin states, tRNAs are associated with transcriptionally active regions

The Chromatin State Segmentation by HMM from ENCODE/Broad covers a spectrum of chromatin states, ranging from active promoters and strong enhancers to heterochromatin and repetitive/copy number variation regions. Expectedly, we found nearly symmetrical distribution of enrichment/depletion of ncRNA associations in opposing chromatin states, reflecting their presence in either active or repressed regions and, conversely, absence in the region with opposite regulatory role. Again, piRNA and tRNA classes showed greater range of p-values (Figure 4B).

piRNAs were strongly enriched in the repressed chromatin (evidence from 9 cell lines), heterochromatin (5 cell lines) and insulator (3 cell lines) regions, as well as in the repetitive/CNV regions in the H1hESC cell line. Conversely, they were depleted in the active, weak and poised promoters (9, 8 and 4 cell lines, respectively), transcription elongation and transition regions (9 and 7 cell lines, respectively), strong and weak enhancers (11 cell lines) (Figure 5C, Additional files 4, 5). Although these findings suggest piRNAs are generally located at the inactive regions of the genome, cell type-specific chromatin states also showed an exception from these observations. For example, piRNAs were depleted in the heterochromatin regions in only the K562 cell line.

tRNAs showed greater diversity in cell type-specific enriched and depleted associations with chromatin states. They were enriched in the active, weak, and poised promoters (9, 9, and 6 cell lines, respectively), and depleted in heterochromatin regions in 6 cell lines. Although this suggests active transcription processes associated with tRNAs, they were depleted in transcription elongation, transition, weak transcription (9, 8, and 6 cell lines, respectively), and in weak enhancers in the HMEC and HUVEC cell lines. tRNAs were also strongly overrepresented in the repetitive/CNV regions in 9 cell lines (Figure 5C, Additional files 4, 5).

Other ncRNA classes showed similar enrichment/depletion patterns in the antagonistic chromatin state regions. Similar to the histone marks, chromatin states were the major driving force behind clustering. For example, pri-miRNA, miRNA, and lncRNA classes were depleted in heterochromatin regions in the majority of the cell lines, while rRNA, 7SK and spliceosomal classes were enriched in these regions (Figure 5D, Additional files 4, 6).

The snoRNA class, and its members C/D box, H/ACA box and scaRNAs were enriched in the "transcription elongation" and "transcription transition" regions in H1hESC cells, and depleted in the "repressed" regions in NHEK and HepG2 cells. These classes were also enriched in the "strong enhancers" and depleted in "weak transcription" chromatin regions in the K562 cell line. The Y-RNA and SRP-RNA classes, on the contrary, were enriched in weak transcription regions in K562 cells (Figure 5D, Additional files 4, 6).

### piRNAs are depleted in, while tRNAs are strongly enriched in, transcription factor binding sites

tRNAs showed strong enrichment in their tendencies to co-localize with 70 TFBSs (p < 0.01, adjusted for multiple testing) (Figure 4C, 5E, Additional files 4, 5). Not unexpectedly, the strongest association was RPC155 (p.adj = 4.43E-89), a catalytic core and the largest (155kDa) component of RNA polymerase III (Pol III), which synthesizes tRNAs as well as small RNAs, such as 5S rRNA. Other Pol III components were also enriched, such as BDP1 (B double prime 1, subunit of Pol III transcription initiation factor IIIB, p.adj = 2.85E-72), TFIIIC-110 (a 110kDa subunit of the Pol III that initiates assembly of the transcription complex on tRNA, p.adj = 9.27E-70), BRF1 (B-related factor 1, a subunit of the Pol III playing in transcription initiation on genes encoding tRNAs, 5S rRNAs, and other small structural RNAs, p.adj = 2.12E-48), Pol3 (aka RPC32, involved either in the recruitment and stabilization of the subcomplex within Pol III, or in stimulating catalytic function of other subunits during initiation, p.adj = 5.28E-34). TBP, TATA-binding protein, a subunit of the transcription factor TFIID (p.adj = 8.19E-80) has also been shown to participate in the initiation complex assembly on tRNA [36]. A general co-activator protein p300 (aka EP300 of E1A binding protein p300, p.adj = 2.01E-65) was also strongly associated with tRNAs.

Pol III can sense non-self dsDNA that serves as template for transcription into dsRNA. Such non-self Pol III transcripts induce type I interferon and NF-kB through RIG-I pathway [37,38]. We observed strong enrichment of immune-related transcription factor binding sites associated with tRNAs. Notably, ETS1, a transcription factor involved in hematopoietic cell differentiation and the development of lymphoid tissues, was the most strongly enriched (p.adj = 8.72E-38), accompanied by ELF1 (aka E74-like factor 1, ets domain transcription factor, p.adj = 5.71E-30). Other well known immune-related transcription factors include NFkB, IRF1, IRF4, and STAT1 (Additional file 5).

piRNAs showed completely the opposite picture (Figure 4C, 5E), being statistically significantly depleted in 59 TFBSs at p-value adjusted for multiple testing <0.01 (Additional files 4, 5). piRNAs were strongly depleted in Pol2 binding sites assessed by four different antibodies (Pol2, p.adj = 1.50E-22; Pol2-4H8, p.adj = 2.55E-18; Pol2(b), p.adj = 5.67E-09; Pol2(phosphoS2), p.adj = 1.03E-06). We also observed the opposite behavior of the Pol III-related proteins, with TBP (p.adj = 5.94E-16), RCP155 (p.adj = 1.41E-12), BDP1 (p.adj = 7.74E-10), TFIIIC-110 (p.adj = 1.38E-08), Pol3 (p.adj = 1.67E-04), and p300 (p.adj = 5.30E-13) binding sites.

The aforementioned RNA polymerase II- and III-related TFBSs were also statistically significantly associated with other ncRNA classes (Figure 5F, Additional files 4, 6). A general trend was that an ncRNA class shows opposite associations with RNA polymerases II and III, being generally associated with Pol II sites and depleted in Pol III-related sites. miRNAs were found enriched in Pol II sites, which corroborates earlier reports [39].

## Discussion

We comprehensively investigated statistically significant genomic associations between a total of 15 ncRNA classes versus 420 cell type-specific epigenomic elements. Although the ENCODE project provides cell type-specific information about epigenomic elements, we generally did not observe preferential cell type specificity of the enrichments. Epigenomic elements associated with different ncRNA classes were generally similarly enriched across multiple cell lines and were the driving force behind clustering patterns confirming that epigenomic relationships hold across multiple cell lines [40]. This can be illustrated by similar patterns of enrichment among spliceosomal, rRNA and 7SK ncRNAs in the heterochromatin regions in multiple cell lines (Figure 5D), association of C/D box and snoRNAs with H3K79me2 mark across several cell lines (Figure 5B), and by similar observations in other ncRNA classes (Additional files 4, 5, 6). There were exceptions from this general trend; for example, piRNAs were strongly enriched in the heterochromatin regions in all but the K562 cell line, which instead showed statistically significant underrepresentation. Y-RNA and SRP-RNA classes were strongly associated with weak transcription regions exclusively in the K562 cell line. Therefore, we kept the cell type-specific results of our analysis (Additional files 1, 2) while describing our results from the epigenomic point of view, mentioning cell line specificity only where appropriate.

Our study revealed both expected (positive controls) and novel relationships among ncRNA classes and epigenomic elements. For example, C/D box, H/ACAbox, and snoRNA were grouped by their common epigenomic background, being subsets of a larger snRNA class. Yet, the relationship between long non-coding RNAs and miRNAs is interesting, as they are located in different genomic regions but shared similar epigenomic background. Both lncRNA and miRNA classes were depleted in the heterochromatin regions in multiple cell lines, were located in the poised promoter regions in the H1hESC cell line, and showed similar enrichment in several histone modification marks (Additional files 4, 6). Yet they were differentially associated with active promoters, with only lncRNAs being associated with these regions in the HSMM and HUVEC cell lines. The lncRNA class was also enriched in the repressed regions of the genome in GM12878 and NHEK cell lines, which is consistent with an observation that >20% of lncRNAs are bound by the Polycomb Repressive Complex 2 (PRC2) [41], with miRNAs showing no measurable enrichment. miRNAs, on the contrary, were enriched in the transcription elongation regions in HMEC, HUVEC, and NHLF cell lines. miRNAs were depleted in the Pol III transcription factor binding regions, and enriched only in the Pol2 binding sites, while lncRNAs did not show any associations with any of these regions. Instead, lncRNAs were enriched in SUZ12 binding sites, consistent with an observation that PRC2-transcribed smaller ~50-200 nt RNAs that interact with SUZ12 to mediate gene repression [42].

We observed strong statistically significant associations of the tRNA and piRNA classes of non-coding RNAs with epigenomic elements. This can't be attributed solely to the size of these classes, since ncRNA classes having more members did not show such enrichment diversity. The mean length of tRNAs and piRNAs were among the smallest among all ncRNA classes (Table 1), thus diminishing the probability of overlapping with epigenomic elements on a per-transcript basis. Yet, the significance of the identified associations suggests strong epigenomic regulation of tRNA and piRNA. We expect our analyses to produce more focused results as the ncRNA classes will become better defined with more members showing clearer enrichment patterns. This is especially important for small ncRNA classes, like scaRNAs, which did not have sufficient numbers to demonstrate statistically significant enrichments. Advancements of the ENCODE project with more epigenomic data being available will further aid analysis of cell type-specific associations.

Our enrichment analysis has limitations. We define ncRNA classes by the genomic coordinates of ncRNA elements, and test these locations for the enriched co-localizations with epigenomic elements. Thus, our enrichment analysis of transcription factor binding sites does not consider the promoter regions of the ncRNAs or, if they don't have promoters, then their local cis-regulatory genomic environment. Yet, the transcription of ncRNAs can be different from that of genes, with ncRNAs often originating from exons and introns [16,43], promoters of the genes [18], or intergenic regions by mechanisms apparently different from canonical transcription factor binding [12]. Transcription factors, on the other hand, show a great variety of binding patterns, being located within gene bodies, overlapping first exons, and occurring in the intergenic regions [44]. Our analysis revealed TF binding preference (or the absence of it) within or partially overlapping ncRNA bodies without making assumptions about the mechanism of ncRNA biogenesis. While the full potential of transcription factor co-localization with ncRNA classes remains to be tested experimentally, we are confident our findings of differential transcription factor co-localization with ncRNAs will reveal novel relationships among non-coding and coding parts of the genome.

In the current study we did not consider the amount of overlap of ncRNAs with epigenomic elements. While this is not critical for the analysis of, for example, single nucleotide polymorphisms with length equal to 1 nt, ncRNAs may show a variety of co-localization patterns, ranging from being located within an epigenomic element to overlapping it completely or partially. We restricted our analysis by considering any overlap as co-localization for computational efficiency and ease of interpretation. However, our future work will include comparison of the percent of overlap observed for an ncRNA class vs. that of for a random selection. This "overlap enrichment" analysis may provide better resolution of spatial relationships of ncRNA classes with epigenomic elements.

## Conclusions

We present the use of our method for finding statistically significant relationships between experimental regions of interest (ncRNA classes in the current study) with cell type-specific epigenomic data from the ENCODE project (such as transcription factor binding sites, histone modification marks, chromatin segmentation states). We demonstrate the utility of statistically significant epigenomic associations to classify and contrast ncRNA classes, and to outline their potential functional roles and mechanisms of biogenesis from epigenomic perspective. Our method, implemented as the open source software GenomeRunner (http://sourceforge.net/projects/genomerunner/) [28] can provide interpretation of any experimental genome-wide data within the growing amount of epigenomic data from the ENCODE project [29].

## Availability

GenomeRunner, the main method used in this work, is available at http://sourceforge.net/projects/genomerunner

## Competing interests

The authors declare that they have no competing interests.

## Authors' contributions

MGD conceived the idea of using epigenomic elements for systematic ncRNA classification, designed and conducted the experiments, interpreted the results, drafted the manuscript and prepared it for submission. CBG helped with data analysis and generation of the results. KAK helped with generation of the results. JDW helped with interpretation of the results and writing the manuscript. All authors read and approved the final manuscript.

## Supplementary Material

Additional file 1 The ENCODE data summary used in the current study Click here for file

Additional file 2 **Cell types used to obtain cell type-specific epigenomic elements**. The vocabulary is extracted from the ENCODE Consortium Data Coordination Center at UCSC (http://genome.ucsc.edu/ENCODE/cellTypes.html). M/F/U indicate cell/tissue donor was of male, female, or unknown gender, respectively. Click here for file

Additional file 3 Brief description of the known roles of histone modification marks Click here for file

Additional file 4 **Enrichment matrix for the different ncRNA classes**. Rows show cell type specific epigenomic marks, except for the transcription factor binding sites (data combined from multiple cell lines). -log10(p.adjusted for multiple testing) values are shown. Red font highlights epigenomic marks enriched at p.adj < 0.01. Green font and a "-" sign highlight depleted epigenomic marks at p.adj < 0.01. Rows containing no statistically significant values in at least one ncRNA class are removed for clarity. Click here for file

Additional file 5 **Epigenomic elements statistically significantly associated with tRNA and piRNA classes**. Regular & red font indicates enriched associations, italics & gree indicates depleted associations. Common transcription factors for both tRNA and piRNA classes are not colored, as they show enriched associations with tRNA and depleted associations with piRNA classes. Click here for file

Additional file 6 **Epigenomic elements statistically significantly associated with ncRNA classes other that tRNA and piRNA classes**. Regular & red font indicates enriched associations, italics & green indicates depleted associations. Click here for file

## Declarations

Publication of this article is funded in part by the National Institutes of Health (NIH, grants # 1P20GM103636 and 8P20GM103456 to JDW), the National Institute of Arthritis and Musculoskeletal and Skin Diseases (a subaward from grant # P30 AR053483 to MGD), and an Institutional Development Award (IDeA) from the National Institute of General Medical Sciences (a subaward from grant # P30GM103510 to MGD). The content of this article is solely the responsibility of the authors and does not necessarily represent the official views of the National Institutes of Health.

This article has been published as part of *BMC Bioinformatics *Volume 14 Supplement 14, 2013: Proceedings of the Tenth Annual MCBIOS Conference. Discovery in a sea of data. The full contents of the supplement are available online at http://www.biomedcentral.com/bmcbioinformatics/supplements/14/S14.
