# Supplementary material for: Systematic classification of non-coding RNAs by epigenomic similarity
Source: BMC Bioinformatics. 2013 Oct 9;14(Suppl 14):S2. doi: 10.1186/1471-2105-14-S14-S2 (PMC3851203; doi:10.1186/1471-2105-14-S14-S2)
Supplement: Additional file 1 — The ENCODE data summary used in the current study [file 1471-2105-14-S14-S2-S1.docx]

**Supplemental Table 1.** The ENCODE data summary used in the current study.

| **Data source / Track prefix** | **Data description** | **Data link** |
| --- | --- | --- |
| wgEncodeBroadHmm… | Chromatin State Segmentation by HMM from ENCODE/Broad | <http://genome.ucsc.edu/cgi-bin/hgTrackUi?hgsid=334027771&g=wgEncodeBroadHmm> |
| wgEncodeBroadHistone… | Histone Modifications by ChIP-seq from ENCODE/Broad Institute | <http://genome.ucsc.edu/cgi-bin/hgTrackUi?hgsid=334027771&g=wgEncodeBroadHistone> |
| wgEncodeRegTfbsClusteredV2 | Transcription Factor ChIP-seq from ENCODE | <http://genome.ucsc.edu/cgi-bin/hgTrackUi?hgsid=334027771&g=wgEncodeRegTfbsClusteredV2> |

| **Track Name** | **Cell line** | **Epigenomic element** |
| --- | --- | --- |
| wgEncodeBroadHmmGm12878HMM | GM12878 | 1_Active_Promoter |
| wgEncodeBroadHmmGm12878HMM | GM12878 | 2_Weak_Promoter |
| wgEncodeBroadHmmGm12878HMM | GM12878 | 3_Poised_Promoter |
| wgEncodeBroadHmmGm12878HMM | GM12878 | 4_Strong_Enhancer |
| wgEncodeBroadHmmGm12878HMM | GM12878 | 5_Strong_Enhancer |
| wgEncodeBroadHmmGm12878HMM | GM12878 | 6_Weak_Enhancer |
| wgEncodeBroadHmmGm12878HMM | GM12878 | 7_Weak_Enhancer |
| wgEncodeBroadHmmGm12878HMM | GM12878 | 8_Insulator |
| wgEncodeBroadHmmGm12878HMM | GM12878 | 9_Txn_Transition |
| wgEncodeBroadHmmGm12878HMM | GM12878 | 10_Txn_Elongation |
| wgEncodeBroadHmmGm12878HMM | GM12878 | 11_Weak_Txn |
| wgEncodeBroadHmmGm12878HMM | GM12878 | 12_Repressed |
| wgEncodeBroadHmmGm12878HMM | GM12878 | 13_Heterochrom/lo |
| wgEncodeBroadHmmGm12878HMM | GM12878 | 14_Repetitive/CNV |
| wgEncodeBroadHmmGm12878HMM | GM12878 | 15_Repetitive/CNV |
| wgEncodeBroadHmmH1hescHMM | H1hESC | 1_Active_Promoter |
| wgEncodeBroadHmmH1hescHMM | H1hESC | 2_Weak_Promoter |
| wgEncodeBroadHmmH1hescHMM | H1hESC | 3_Poised_Promoter |
| wgEncodeBroadHmmH1hescHMM | H1hESC | 4_Strong_Enhancer |
| wgEncodeBroadHmmH1hescHMM | H1hESC | 5_Strong_Enhancer |
| wgEncodeBroadHmmH1hescHMM | H1hESC | 6_Weak_Enhancer |
| wgEncodeBroadHmmH1hescHMM | H1hESC | 7_Weak_Enhancer |
| wgEncodeBroadHmmH1hescHMM | H1hESC | 8_Insulator |
| wgEncodeBroadHmmH1hescHMM | H1hESC | 9_Txn_Transition |
| wgEncodeBroadHmmH1hescHMM | H1hESC | 10_Txn_Elongation |
| wgEncodeBroadHmmH1hescHMM | H1hESC | 11_Weak_Txn |
| wgEncodeBroadHmmH1hescHMM | H1hESC | 12_Repressed |
| wgEncodeBroadHmmH1hescHMM | H1hESC | 13_Heterochrom/lo |
| wgEncodeBroadHmmH1hescHMM | H1hESC | 14_Repetitive/CNV |
| wgEncodeBroadHmmH1hescHMM | H1hESC | 15_Repetitive/CNV |
| wgEncodeBroadHmmHepg2HMM | HepG2 | 1_Active_Promoter |
| wgEncodeBroadHmmHepg2HMM | HepG2 | 2_Weak_Promoter |
| wgEncodeBroadHmmHepg2HMM | HepG2 | 3_Poised_Promoter |
| wgEncodeBroadHmmHepg2HMM | HepG2 | 4_Strong_Enhancer |
| wgEncodeBroadHmmHepg2HMM | HepG2 | 5_Strong_Enhancer |
| wgEncodeBroadHmmHepg2HMM | HepG2 | 6_Weak_Enhancer |
| wgEncodeBroadHmmHepg2HMM | HepG2 | 7_Weak_Enhancer |
| wgEncodeBroadHmmHepg2HMM | HepG2 | 8_Insulator |
| wgEncodeBroadHmmHepg2HMM | HepG2 | 9_Txn_Transition |
| wgEncodeBroadHmmHepg2HMM | HepG2 | 10_Txn_Elongation |
| wgEncodeBroadHmmHepg2HMM | HepG2 | 11_Weak_Txn |
| wgEncodeBroadHmmHepg2HMM | HepG2 | 12_Repressed |
| wgEncodeBroadHmmHepg2HMM | HepG2 | 13_Heterochrom/lo |
| wgEncodeBroadHmmHepg2HMM | HepG2 | 14_Repetitive/CNV |
| wgEncodeBroadHmmHepg2HMM | HepG2 | 15_Repetitive/CNV |
| wgEncodeBroadHmmHmecHMM | Hmec | 1_Active_Promoter |
| wgEncodeBroadHmmHmecHMM | Hmec | 2_Weak_Promoter |
| wgEncodeBroadHmmHmecHMM | Hmec | 3_Poised_Promoter |
| wgEncodeBroadHmmHmecHMM | Hmec | 4_Strong_Enhancer |
| wgEncodeBroadHmmHmecHMM | Hmec | 5_Strong_Enhancer |
| wgEncodeBroadHmmHmecHMM | Hmec | 6_Weak_Enhancer |
| wgEncodeBroadHmmHmecHMM | Hmec | 7_Weak_Enhancer |
| wgEncodeBroadHmmHmecHMM | Hmec | 8_Insulator |
| wgEncodeBroadHmmHmecHMM | Hmec | 9_Txn_Transition |
| wgEncodeBroadHmmHmecHMM | Hmec | 10_Txn_Elongation |
| wgEncodeBroadHmmHmecHMM | Hmec | 11_Weak_Txn |
| wgEncodeBroadHmmHmecHMM | Hmec | 12_Repressed |
| wgEncodeBroadHmmHmecHMM | Hmec | 13_Heterochrom/lo |
| wgEncodeBroadHmmHmecHMM | Hmec | 14_Repetitive/CNV |
| wgEncodeBroadHmmHmecHMM | Hmec | 15_Repetitive/CNV |
| wgEncodeBroadHmmHsmmHMM | Hsmm | 1_Active_Promoter |
| wgEncodeBroadHmmHsmmHMM | Hsmm | 2_Weak_Promoter |
| wgEncodeBroadHmmHsmmHMM | Hsmm | 3_Poised_Promoter |
| wgEncodeBroadHmmHsmmHMM | Hsmm | 4_Strong_Enhancer |
| wgEncodeBroadHmmHsmmHMM | Hsmm | 5_Strong_Enhancer |
| wgEncodeBroadHmmHsmmHMM | Hsmm | 6_Weak_Enhancer |
| wgEncodeBroadHmmHsmmHMM | Hsmm | 7_Weak_Enhancer |
| wgEncodeBroadHmmHsmmHMM | Hsmm | 8_Insulator |
| wgEncodeBroadHmmHsmmHMM | Hsmm | 9_Txn_Transition |
| wgEncodeBroadHmmHsmmHMM | Hsmm | 10_Txn_Elongation |
| wgEncodeBroadHmmHsmmHMM | Hsmm | 11_Weak_Txn |
| wgEncodeBroadHmmHsmmHMM | Hsmm | 12_Repressed |
| wgEncodeBroadHmmHsmmHMM | Hsmm | 13_Heterochrom/lo |
| wgEncodeBroadHmmHsmmHMM | Hsmm | 14_Repetitive/CNV |
| wgEncodeBroadHmmHsmmHMM | Hsmm | 15_Repetitive/CNV |
| wgEncodeBroadHmmHuvecHMM | Huvec | 1_Active_Promoter |
| wgEncodeBroadHmmHuvecHMM | Huvec | 2_Weak_Promoter |
| wgEncodeBroadHmmHuvecHMM | Huvec | 3_Poised_Promoter |
| wgEncodeBroadHmmHuvecHMM | Huvec | 4_Strong_Enhancer |
| wgEncodeBroadHmmHuvecHMM | Huvec | 5_Strong_Enhancer |
| wgEncodeBroadHmmHuvecHMM | Huvec | 6_Weak_Enhancer |
| wgEncodeBroadHmmHuvecHMM | Huvec | 7_Weak_Enhancer |
| wgEncodeBroadHmmHuvecHMM | Huvec | 8_Insulator |
| wgEncodeBroadHmmHuvecHMM | Huvec | 9_Txn_Transition |
| wgEncodeBroadHmmHuvecHMM | Huvec | 10_Txn_Elongation |
| wgEncodeBroadHmmHuvecHMM | Huvec | 11_Weak_Txn |
| wgEncodeBroadHmmHuvecHMM | Huvec | 12_Repressed |
| wgEncodeBroadHmmHuvecHMM | Huvec | 13_Heterochrom/lo |
| wgEncodeBroadHmmHuvecHMM | Huvec | 14_Repetitive/CNV |
| wgEncodeBroadHmmHuvecHMM | Huvec | 15_Repetitive/CNV |
| wgEncodeBroadHmmK562HMM | K562 | 1_Active_Promoter |
| wgEncodeBroadHmmK562HMM | K562 | 2_Weak_Promoter |
| wgEncodeBroadHmmK562HMM | K562 | 3_Poised_Promoter |
| wgEncodeBroadHmmK562HMM | K562 | 4_Strong_Enhancer |
| wgEncodeBroadHmmK562HMM | K562 | 5_Strong_Enhancer |
| wgEncodeBroadHmmK562HMM | K562 | 6_Weak_Enhancer |
| wgEncodeBroadHmmK562HMM | K562 | 7_Weak_Enhancer |
| wgEncodeBroadHmmK562HMM | K562 | 8_Insulator |
| wgEncodeBroadHmmK562HMM | K562 | 9_Txn_Transition |
| wgEncodeBroadHmmK562HMM | K562 | 10_Txn_Elongation |
| wgEncodeBroadHmmK562HMM | K562 | 11_Weak_Txn |
| wgEncodeBroadHmmK562HMM | K562 | 12_Repressed |
| wgEncodeBroadHmmK562HMM | K562 | 13_Heterochrom/lo |
| wgEncodeBroadHmmK562HMM | K562 | 14_Repetitive/CNV |
| wgEncodeBroadHmmK562HMM | K562 | 15_Repetitive/CNV |
| wgEncodeBroadHmmNhekHMM | Nhek | 1_Active_Promoter |
| wgEncodeBroadHmmNhekHMM | Nhek | 2_Weak_Promoter |
| wgEncodeBroadHmmNhekHMM | Nhek | 3_Poised_Promoter |
| wgEncodeBroadHmmNhekHMM | Nhek | 4_Strong_Enhancer |
| wgEncodeBroadHmmNhekHMM | Nhek | 5_Strong_Enhancer |
| wgEncodeBroadHmmNhekHMM | Nhek | 6_Weak_Enhancer |
| wgEncodeBroadHmmNhekHMM | Nhek | 7_Weak_Enhancer |
| wgEncodeBroadHmmNhekHMM | Nhek | 8_Insulator |
| wgEncodeBroadHmmNhekHMM | Nhek | 9_Txn_Transition |
| wgEncodeBroadHmmNhekHMM | Nhek | 10_Txn_Elongation |
| wgEncodeBroadHmmNhekHMM | Nhek | 11_Weak_Txn |
| wgEncodeBroadHmmNhekHMM | Nhek | 12_Repressed |
| wgEncodeBroadHmmNhekHMM | Nhek | 13_Heterochrom/lo |
| wgEncodeBroadHmmNhekHMM | Nhek | 14_Repetitive/CNV |
| wgEncodeBroadHmmNhekHMM | Nhek | 15_Repetitive/CNV |
| wgEncodeBroadHmmNhlfHMM | Nhlf | 1_Active_Promoter |
| wgEncodeBroadHmmNhlfHMM | Nhlf | 2_Weak_Promoter |
| wgEncodeBroadHmmNhlfHMM | Nhlf | 3_Poised_Promoter |
| wgEncodeBroadHmmNhlfHMM | Nhlf | 4_Strong_Enhancer |
| wgEncodeBroadHmmNhlfHMM | Nhlf | 5_Strong_Enhancer |
| wgEncodeBroadHmmNhlfHMM | Nhlf | 6_Weak_Enhancer |
| wgEncodeBroadHmmNhlfHMM | Nhlf | 7_Weak_Enhancer |
| wgEncodeBroadHmmNhlfHMM | Nhlf | 8_Insulator |
| wgEncodeBroadHmmNhlfHMM | Nhlf | 9_Txn_Transition |
| wgEncodeBroadHmmNhlfHMM | Nhlf | 10_Txn_Elongation |
| wgEncodeBroadHmmNhlfHMM | Nhlf | 11_Weak_Txn |
| wgEncodeBroadHmmNhlfHMM | Nhlf | 12_Repressed |
| wgEncodeBroadHmmNhlfHMM | Nhlf | 13_Heterochrom/lo |
| wgEncodeBroadHmmNhlfHMM | Nhlf | 14_Repetitive/CNV |
| wgEncodeBroadHmmNhlfHMM | Nhlf | 15_Repetitive/CNV |
| wgEncodeBroadHistoneGm12878CtcfStdPk | Gm12878 | Ctcf |
| wgEncodeBroadHistoneGm12878H2azStdPk | Gm12878 | H2az |
| wgEncodeBroadHistoneGm12878H3k27acStdPk | Gm12878 | H3k27ac |
| wgEncodeBroadHistoneGm12878H3k27me3StdPk | Gm12878 | H3k27me3 |
| wgEncodeBroadHistoneGm12878H3k36me3StdPk | Gm12878 | H3k36me3 |
| wgEncodeBroadHistoneGm12878H3k4me1StdPk | Gm12878 | H3k4me1 |
| wgEncodeBroadHistoneGm12878H3k4me2StdPk | Gm12878 | H3k4me2 |
| wgEncodeBroadHistoneGm12878H3k4me3StdPk | Gm12878 | H3k4me3 |
| wgEncodeBroadHistoneGm12878H3k79me2StdPk | Gm12878 | H3k79me2 |
| wgEncodeBroadHistoneGm12878H3k9acStdPk | Gm12878 | H3k9ac |
| wgEncodeBroadHistoneGm12878H3k9me3StdPk | Gm12878 | H3k9me3 |
| wgEncodeBroadHistoneGm12878H4k20me1StdPk | Gm12878 | H4k20me1 |
| wgEncodeBroadHistoneH1hescCtcfStdPk | H1hesc | Ctcf |
| wgEncodeBroadHistoneH1hescH3k27acStdPk | H1hesc | H3k27ac |
| wgEncodeBroadHistoneH1hescH3k27me3StdPk | H1hesc | H3k27me3 |
| wgEncodeBroadHistoneH1hescH3k36me3StdPk | H1hesc | H3k36me3 |
| wgEncodeBroadHistoneH1hescH3k4me1StdPk | H1hesc | H3k4me1 |
| wgEncodeBroadHistoneH1hescH3k4me2StdPk | H1hesc | H3k4me2 |
| wgEncodeBroadHistoneH1hescH3k4me3StdPk | H1hesc | H3k4me3 |
| wgEncodeBroadHistoneH1hescH3k9acStdPk | H1hesc | H3k9ac |
| wgEncodeBroadHistoneH1hescH4k20me1StdPk | H1hesc | H4k20me1 |
| wgEncodeBroadHistoneHelas3CtcfStdPk | Helas3 | Ctcf |
| wgEncodeBroadHistoneHelas3H3k27acStdPk | Helas3 | H3k27ac |
| wgEncodeBroadHistoneHelas3H3k27me3StdPk | Helas3 | H3k27me3 |
| wgEncodeBroadHistoneHelas3H3k36me3StdPk | Helas3 | H3k36me3 |
| wgEncodeBroadHistoneHelas3H3k4me2StdPk | Helas3 | H3k4me2 |
| wgEncodeBroadHistoneHelas3H3k4me3StdPk | Helas3 | H3k4me3 |
| wgEncodeBroadHistoneHelas3H3k79me2StdPk | Helas3 | H3k79me2 |
| wgEncodeBroadHistoneHelas3H3k9acStdPk | Helas3 | H3k9ac |
| wgEncodeBroadHistoneHelas3H4k20me1StdPk | Helas3 | H4k20me1 |
| wgEncodeBroadHistoneHelas3Pol2bStdPk | Helas3 | Pol2b |
| wgEncodeBroadHistoneHepg2CtcfStdPk | Hepg2 | Ctcf |
| wgEncodeBroadHistoneHepg2H2azStdPk | Hepg2 | H2az |
| wgEncodeBroadHistoneHepg2H3k27acStdPk | Hepg2 | H3k27ac |
| wgEncodeBroadHistoneHepg2H3k27me3StdPk | Hepg2 | H3k27me3 |
| wgEncodeBroadHistoneHepg2H3k36me3StdPk | Hepg2 | H3k36me3 |
| wgEncodeBroadHistoneHepg2H3k4me2StdPk | Hepg2 | H3k4me2 |
| wgEncodeBroadHistoneHepg2H3k4me3StdPk | Hepg2 | H3k4me3 |
| wgEncodeBroadHistoneHepg2H3k79me2StdPk | Hepg2 | H3k79me2 |
| wgEncodeBroadHistoneHepg2H3k9acStdPk | Hepg2 | H3k9ac |
| wgEncodeBroadHistoneHepg2H4k20me1StdPk | Hepg2 | H4k20me1 |
| wgEncodeBroadHistoneHmecCtcfStdPk | Hmec | Ctcf |
| wgEncodeBroadHistoneHmecH3k27acStdPk | Hmec | H3k27ac |
| wgEncodeBroadHistoneHmecH3k27me3StdPk | Hmec | H3k27me3 |
| wgEncodeBroadHistoneHmecH3k36me3StdPk | Hmec | H3k36me3 |
| wgEncodeBroadHistoneHmecH3k4me1StdPk | Hmec | H3k4me1 |
| wgEncodeBroadHistoneHmecH3k4me2StdPk | Hmec | H3k4me2 |
| wgEncodeBroadHistoneHmecH3k4me3StdPk | Hmec | H3k4me3 |
| wgEncodeBroadHistoneHmecH3k9acStdPk | Hmec | H3k9ac |
| wgEncodeBroadHistoneHmecH4k20me1StdPk | Hmec | H4k20me1 |
| wgEncodeBroadHistoneHsmmCtcfStdPk | Hsmm | Ctcf |
| wgEncodeBroadHistoneHsmmH2azStdPk | Hsmm | H2az |
| wgEncodeBroadHistoneHsmmH3k27acStdPk | Hsmm | H3k27ac |
| wgEncodeBroadHistoneHsmmH3k27me3StdPk | Hsmm | H3k27me3 |
| wgEncodeBroadHistoneHsmmH3k36me3StdPk | Hsmm | H3k36me3 |
| wgEncodeBroadHistoneHsmmH3k4me1StdPk | Hsmm | H3k4me1 |
| wgEncodeBroadHistoneHsmmH3k4me2StdPk | Hsmm | H3k4me2 |
| wgEncodeBroadHistoneHsmmH3k4me3StdPk | Hsmm | H3k4me3 |
| wgEncodeBroadHistoneHsmmH3k79me2StdPk | Hsmm | H3k79me2 |
| wgEncodeBroadHistoneHsmmH3k9acStdPk | Hsmm | H3k9ac |
| wgEncodeBroadHistoneHsmmH3k9me3StdPk | Hsmm | H3k9me3 |
| wgEncodeBroadHistoneHsmmH4k20me1StdPk | Hsmm | H4k20me1 |
| wgEncodeBroadHistoneHsmmtCtcfStdPk | Hsmmt | Ctcf |
| wgEncodeBroadHistoneHsmmtH2azStdPk | Hsmmt | H2az |
| wgEncodeBroadHistoneHsmmtH3k27acStdPk | Hsmmt | H3k27ac |
| wgEncodeBroadHistoneHsmmtH3k36me3StdPk | Hsmmt | H3k36me3 |
| wgEncodeBroadHistoneHsmmtH3k4me1StdPk | Hsmmt | H3k4me1 |
| wgEncodeBroadHistoneHsmmtH3k4me2StdPk | Hsmmt | H3k4me2 |
| wgEncodeBroadHistoneHsmmtH3k4me3StdPk | Hsmmt | H3k4me3 |
| wgEncodeBroadHistoneHsmmtH3k79me2StdPk | Hsmmt | H3k79me2 |
| wgEncodeBroadHistoneHsmmtH3k9acStdPk | Hsmmt | H3k9ac |
| wgEncodeBroadHistoneHsmmtH4k20me1StdPk | Hsmmt | H4k20me1 |
| wgEncodeBroadHistoneHuvecCtcfStdPk | Huvec | Ctcf |
| wgEncodeBroadHistoneHuvecH3k27acStdPk | Huvec | H3k27ac |
| wgEncodeBroadHistoneHuvecH3k27me3StdPk | Huvec | H3k27me3 |
| wgEncodeBroadHistoneHuvecH3k36me3StdPk | Huvec | H3k36me3 |
| wgEncodeBroadHistoneHuvecH3k4me1StdPk | Huvec | H3k4me1 |
| wgEncodeBroadHistoneHuvecH3k4me2StdPk | Huvec | H3k4me2 |
| wgEncodeBroadHistoneHuvecH3k4me3StdPk | Huvec | H3k4me3 |
| wgEncodeBroadHistoneHuvecH3k9acStdPk | Huvec | H3k9ac |
| wgEncodeBroadHistoneHuvecH3k9me1StdPk | Huvec | H3k9me1 |
| wgEncodeBroadHistoneHuvecH4k20me1StdPk | Huvec | H4k20me1 |
| wgEncodeBroadHistoneHuvecPol2bStdPk | Huvec | Pol2b |
| wgEncodeBroadHistoneK562CtcfStdPk | K562 | Ctcf |
| wgEncodeBroadHistoneK562H2azStdPk | K562 | H2az |
| wgEncodeBroadHistoneK562H3k27acStdPk | K562 | H3k27ac |
| wgEncodeBroadHistoneK562H3k27me3StdPk | K562 | H3k27me3 |
| wgEncodeBroadHistoneK562H3k36me3StdPk | K562 | H3k36me3 |
| wgEncodeBroadHistoneK562H3k4me1StdPk | K562 | H3k4me1 |
| wgEncodeBroadHistoneK562H3k4me2StdPk | K562 | H3k4me2 |
| wgEncodeBroadHistoneK562H3k4me3StdPk | K562 | H3k4me3 |
| wgEncodeBroadHistoneK562H3k79me2StdPk | K562 | H3k79me2 |
| wgEncodeBroadHistoneK562H3k9acStdPk | K562 | H3k9ac |
| wgEncodeBroadHistoneK562H3k9me1StdPk | K562 | H3k9me1 |
| wgEncodeBroadHistoneK562H3k9me3StdPk | K562 | H3k9me3 |
| wgEncodeBroadHistoneK562H4k20me1StdPk | K562 | H4k20me1 |
| wgEncodeBroadHistoneK562Pol2bStdPk | K562 | Pol2b |
| wgEncodeBroadHistoneNhaCtcfStdPk | Nha | Ctcf |
| wgEncodeBroadHistoneNhaH3k27acStdPk | Nha | H3k27ac |
| wgEncodeBroadHistoneNhaH3k27me3StdPk | Nha | H3k27me3 |
| wgEncodeBroadHistoneNhaH3k36me3StdPk | Nha | H3k36me3 |
| wgEncodeBroadHistoneNhaH3k4me1StdPk | Nha | H3k4me1 |
| wgEncodeBroadHistoneNhaH3k4me3StdPk | Nha | H3k4me3 |
| wgEncodeBroadHistoneNhdfadCtcfStdPk | Nhdfad | Ctcf |
| wgEncodeBroadHistoneNhdfadH3k27acStdPk | Nhdfad | H3k27ac |
| wgEncodeBroadHistoneNhdfadH3k27me3StdPk | Nhdfad | H3k27me3 |
| wgEncodeBroadHistoneNhdfadH3k36me3StdPk | Nhdfad | H3k36me3 |
| wgEncodeBroadHistoneNhdfadH3k4me2StdPk | Nhdfad | H3k4me2 |
| wgEncodeBroadHistoneNhdfadH3k4me3StdPk | Nhdfad | H3k4me3 |
| wgEncodeBroadHistoneNhdfadH3k9acStdPk | Nhdfad | H3k9ac |
| wgEncodeBroadHistoneNhekCtcfStdPk | Nhek | Ctcf |
| wgEncodeBroadHistoneNhekH3k27acStdPk | Nhek | H3k27ac |
| wgEncodeBroadHistoneNhekH3k27me3StdPk | Nhek | H3k27me3 |
| wgEncodeBroadHistoneNhekH3k36me3StdPk | Nhek | H3k36me3 |
| wgEncodeBroadHistoneNhekH3k4me1StdPk | Nhek | H3k4me1 |
| wgEncodeBroadHistoneNhekH3k4me2StdPk | Nhek | H3k4me2 |
| wgEncodeBroadHistoneNhekH3k4me3StdPk | Nhek | H3k4me3 |
| wgEncodeBroadHistoneNhekH3k9acStdPk | Nhek | H3k9ac |
| wgEncodeBroadHistoneNhekH3k9me1StdPk | Nhek | H3k9me1 |
| wgEncodeBroadHistoneNhekH4k20me1StdPk | Nhek | H4k20me1 |
| wgEncodeBroadHistoneNhekPol2bStdPk | Nhek | Pol2b |
| wgEncodeBroadHistoneNhlfCtcfStdPk | Nhlf | Ctcf |
| wgEncodeBroadHistoneNhlfH3k27acStdPk | Nhlf | H3k27ac |
| wgEncodeBroadHistoneNhlfH3k27me3StdPk | Nhlf | H3k27me3 |
| wgEncodeBroadHistoneNhlfH3k36me3StdPk | Nhlf | H3k36me3 |
| wgEncodeBroadHistoneNhlfH3k4me1StdPk | Nhlf | H3k4me1 |
| wgEncodeBroadHistoneNhlfH3k4me2StdPk | Nhlf | H3k4me2 |
| wgEncodeBroadHistoneNhlfH3k4me3StdPk | Nhlf | H3k4me3 |
| wgEncodeBroadHistoneNhlfH3k9acStdPk | Nhlf | H3k9ac |
| wgEncodeBroadHistoneNhlfH4k20me1StdPk | Nhlf | H4k20me1 |
| wgEncodeBroadHistoneOsteoblCtcfStdPk | Osteobl | Ctcf |
| wgEncodeBroadHistoneOsteoblH2azStdPk | Osteobl | H2az |
| wgEncodeBroadHistoneOsteoblH3k27acStdPk | Osteobl | H3k27ac |
| wgEncodeBroadHistoneOsteoblH3k36me3StdPk | Osteobl | H3k36me3 |
| wgEncodeBroadHistoneOsteoblH3k4me1StdPk | Osteobl | H3k4me1 |
| wgEncodeBroadHistoneOsteoblH3k4me2StdPk | Osteobl | H3k4me2 |
| wgEncodeBroadHistoneOsteoblH3k9me3StdPk | Osteobl | H3k9me3 |
| wgEncodeRegTfbsClusteredV2 | Combined | AP-2alpha |
| wgEncodeRegTfbsClusteredV2 | Combined | AP-2gamma |
| wgEncodeRegTfbsClusteredV2 | Combined | ATF3 |
| wgEncodeRegTfbsClusteredV2 | Combined | BAF155 |
| wgEncodeRegTfbsClusteredV2 | Combined | BAF170 |
| wgEncodeRegTfbsClusteredV2 | Combined | BATF |
| wgEncodeRegTfbsClusteredV2 | Combined | BCL11A |
| wgEncodeRegTfbsClusteredV2 | Combined | BCL3 |
| wgEncodeRegTfbsClusteredV2 | Combined | BCLAF1_(M33-P5B11) |
| wgEncodeRegTfbsClusteredV2 | Combined | BDP1 |
| wgEncodeRegTfbsClusteredV2 | Combined | BHLHE40 |
| wgEncodeRegTfbsClusteredV2 | Combined | BRCA1_(C-1863) |
| wgEncodeRegTfbsClusteredV2 | Combined | BRF1 |
| wgEncodeRegTfbsClusteredV2 | Combined | BRF2 |
| wgEncodeRegTfbsClusteredV2 | Combined | Brg1 |
| wgEncodeRegTfbsClusteredV2 | Combined | CCNT2 |
| wgEncodeRegTfbsClusteredV2 | Combined | CEBPB |
| wgEncodeRegTfbsClusteredV2 | Combined | c-Fos |
| wgEncodeRegTfbsClusteredV2 | Combined | CHD2_(N-1250) |
| wgEncodeRegTfbsClusteredV2 | Combined | c-Jun |
| wgEncodeRegTfbsClusteredV2 | Combined | c-Myc |
| wgEncodeRegTfbsClusteredV2 | Combined | CtBP2 |
| wgEncodeRegTfbsClusteredV2 | Combined | CTCF |
| wgEncodeRegTfbsClusteredV2 | Combined | CTCF_(C-20) |
| wgEncodeRegTfbsClusteredV2 | Combined | CTCF_(SC-5916) |
| wgEncodeRegTfbsClusteredV2 | Combined | CTCFL_(SC-98982) |
| wgEncodeRegTfbsClusteredV2 | Combined | E2F1 |
| wgEncodeRegTfbsClusteredV2 | Combined | E2F4 |
| wgEncodeRegTfbsClusteredV2 | Combined | E2F6 |
| wgEncodeRegTfbsClusteredV2 | Combined | E2F6_(H-50) |
| wgEncodeRegTfbsClusteredV2 | Combined | EBF |
| wgEncodeRegTfbsClusteredV2 | Combined | EBF1_(C-8) |
| wgEncodeRegTfbsClusteredV2 | Combined | eGFP-FOS |
| wgEncodeRegTfbsClusteredV2 | Combined | eGFP-GATA2 |
| wgEncodeRegTfbsClusteredV2 | Combined | eGFP-HDAC8 |
| wgEncodeRegTfbsClusteredV2 | Combined | eGFP-JunB |
| wgEncodeRegTfbsClusteredV2 | Combined | eGFP-JunD |
| wgEncodeRegTfbsClusteredV2 | Combined | eGFP-NR4A1 |
| wgEncodeRegTfbsClusteredV2 | Combined | Egr-1 |
| wgEncodeRegTfbsClusteredV2 | Combined | ELF1_(SC-631) |
| wgEncodeRegTfbsClusteredV2 | Combined | ELK4 |
| wgEncodeRegTfbsClusteredV2 | Combined | ERalpha_a |
| wgEncodeRegTfbsClusteredV2 | Combined | ERRA |
| wgEncodeRegTfbsClusteredV2 | Combined | ETS1 |
| wgEncodeRegTfbsClusteredV2 | Combined | FOSL1_(SC-183) |
| wgEncodeRegTfbsClusteredV2 | Combined | FOSL2 |
| wgEncodeRegTfbsClusteredV2 | Combined | FOXA1_(C-20) |
| wgEncodeRegTfbsClusteredV2 | Combined | FOXA1_(SC-101058) |
| wgEncodeRegTfbsClusteredV2 | Combined | FOXA2_(SC-6554) |
| wgEncodeRegTfbsClusteredV2 | Combined | GABP |
| wgEncodeRegTfbsClusteredV2 | Combined | GATA-1 |
| wgEncodeRegTfbsClusteredV2 | Combined | GATA-2 |
| wgEncodeRegTfbsClusteredV2 | Combined | GATA2_(CG2-96) |
| wgEncodeRegTfbsClusteredV2 | Combined | GATA3_(SC-268) |
| wgEncodeRegTfbsClusteredV2 | Combined | GCN5 |
| wgEncodeRegTfbsClusteredV2 | Combined | GR |
| wgEncodeRegTfbsClusteredV2 | Combined | GRp20 |
| wgEncodeRegTfbsClusteredV2 | Combined | GTF2B |
| wgEncodeRegTfbsClusteredV2 | Combined | GTF2F1_(RAP-74) |
| wgEncodeRegTfbsClusteredV2 | Combined | HA-E2F1 |
| wgEncodeRegTfbsClusteredV2 | Combined | HDAC2_(SC-6296) |
| wgEncodeRegTfbsClusteredV2 | Combined | HEY1 |
| wgEncodeRegTfbsClusteredV2 | Combined | HMGN3 |
| wgEncodeRegTfbsClusteredV2 | Combined | HNF4A |
| wgEncodeRegTfbsClusteredV2 | Combined | HNF4A_(H-171) |
| wgEncodeRegTfbsClusteredV2 | Combined | HNF4G_(SC-6558) |
| wgEncodeRegTfbsClusteredV2 | Combined | HSF1 |
| wgEncodeRegTfbsClusteredV2 | Combined | Ini1 |
| wgEncodeRegTfbsClusteredV2 | Combined | IRF1 |
| wgEncodeRegTfbsClusteredV2 | Combined | IRF3 |
| wgEncodeRegTfbsClusteredV2 | Combined | IRF4_(M-17) |
| wgEncodeRegTfbsClusteredV2 | Combined | JunD |
| wgEncodeRegTfbsClusteredV2 | Combined | KAP1 |
| wgEncodeRegTfbsClusteredV2 | Combined | MafF_(M8194) |
| wgEncodeRegTfbsClusteredV2 | Combined | MafK_(ab50322) |
| wgEncodeRegTfbsClusteredV2 | Combined | MafK_(SC-477) |
| wgEncodeRegTfbsClusteredV2 | Combined | Max |
| wgEncodeRegTfbsClusteredV2 | Combined | MEF2A |
| wgEncodeRegTfbsClusteredV2 | Combined | MEF2C_(SC-13268) |
| wgEncodeRegTfbsClusteredV2 | Combined | Mxi1_(bHLH) |
| wgEncodeRegTfbsClusteredV2 | Combined | NANOG_(SC-33759) |
| wgEncodeRegTfbsClusteredV2 | Combined | NELFe |
| wgEncodeRegTfbsClusteredV2 | Combined | NF-E2 |
| wgEncodeRegTfbsClusteredV2 | Combined | NF-E2_(H-230) |
| wgEncodeRegTfbsClusteredV2 | Combined | NFKB |
| wgEncodeRegTfbsClusteredV2 | Combined | NF-YA |
| wgEncodeRegTfbsClusteredV2 | Combined | NF-YB |
| wgEncodeRegTfbsClusteredV2 | Combined | Nrf1 |
| wgEncodeRegTfbsClusteredV2 | Combined | NRSF |
| wgEncodeRegTfbsClusteredV2 | Combined | OCT2 |
| wgEncodeRegTfbsClusteredV2 | Combined | p300 |
| wgEncodeRegTfbsClusteredV2 | Combined | p300_(F-4) |
| wgEncodeRegTfbsClusteredV2 | Combined | p300_(N-15) |
| wgEncodeRegTfbsClusteredV2 | Combined | PAX5-C20 |
| wgEncodeRegTfbsClusteredV2 | Combined | PAX5-N19 |
| wgEncodeRegTfbsClusteredV2 | Combined | Pbx3 |
| wgEncodeRegTfbsClusteredV2 | Combined | PGC1A |
| wgEncodeRegTfbsClusteredV2 | Combined | Pol2 |
| wgEncodeRegTfbsClusteredV2 | Combined | Pol2(b) |
| wgEncodeRegTfbsClusteredV2 | Combined | Pol2(phosphoS2) |
| wgEncodeRegTfbsClusteredV2 | Combined | Pol2-4H8 |
| wgEncodeRegTfbsClusteredV2 | Combined | Pol3 |
| wgEncodeRegTfbsClusteredV2 | Combined | POU2F2 |
| wgEncodeRegTfbsClusteredV2 | Combined | POU5F1_(SC-9081) |
| wgEncodeRegTfbsClusteredV2 | Combined | PRDM1_(Val90) |
| wgEncodeRegTfbsClusteredV2 | Combined | PU.1 |
| wgEncodeRegTfbsClusteredV2 | Combined | Rad21 |
| wgEncodeRegTfbsClusteredV2 | Combined | RFX5_(N-494) |
| wgEncodeRegTfbsClusteredV2 | Combined | RPC155 |
| wgEncodeRegTfbsClusteredV2 | Combined | RXRA |
| wgEncodeRegTfbsClusteredV2 | Combined | SETDB1 |
| wgEncodeRegTfbsClusteredV2 | Combined | Sin3Ak-20 |
| wgEncodeRegTfbsClusteredV2 | Combined | SIRT6 |
| wgEncodeRegTfbsClusteredV2 | Combined | SIX5 |
| wgEncodeRegTfbsClusteredV2 | Combined | SMC3_(ab9263) |
| wgEncodeRegTfbsClusteredV2 | Combined | SP1 |
| wgEncodeRegTfbsClusteredV2 | Combined | SP2_(SC-643) |
| wgEncodeRegTfbsClusteredV2 | Combined | SPT20 |
| wgEncodeRegTfbsClusteredV2 | Combined | SREBP1 |
| wgEncodeRegTfbsClusteredV2 | Combined | SREBP2 |
| wgEncodeRegTfbsClusteredV2 | Combined | SRF |
| wgEncodeRegTfbsClusteredV2 | Combined | STAT1 |
| wgEncodeRegTfbsClusteredV2 | Combined | STAT2 |
| wgEncodeRegTfbsClusteredV2 | Combined | STAT3 |
| wgEncodeRegTfbsClusteredV2 | Combined | SUZ12 |
| wgEncodeRegTfbsClusteredV2 | Combined | TAF1 |
| wgEncodeRegTfbsClusteredV2 | Combined | TAF7_(SQ-8) |
| wgEncodeRegTfbsClusteredV2 | Combined | TAL1_(SC-12984) |
| wgEncodeRegTfbsClusteredV2 | Combined | TBP |
| wgEncodeRegTfbsClusteredV2 | Combined | TCF12 |
| wgEncodeRegTfbsClusteredV2 | Combined | TCF4 |
| wgEncodeRegTfbsClusteredV2 | Combined | TFIIIC-110 |
| wgEncodeRegTfbsClusteredV2 | Combined | THAP1_(SC-98174) |
| wgEncodeRegTfbsClusteredV2 | Combined | TR4 |
| wgEncodeRegTfbsClusteredV2 | Combined | USF-1 |
| wgEncodeRegTfbsClusteredV2 | Combined | USF1_(SC-8983) |
| wgEncodeRegTfbsClusteredV2 | Combined | USF2 |
| wgEncodeRegTfbsClusteredV2 | Combined | WHIP |
| wgEncodeRegTfbsClusteredV2 | Combined | XRCC4 |
| wgEncodeRegTfbsClusteredV2 | Combined | YY1 |
| wgEncodeRegTfbsClusteredV2 | Combined | YY1_(C-20) |
| wgEncodeRegTfbsClusteredV2 | Combined | ZBTB33 |
| wgEncodeRegTfbsClusteredV2 | Combined | ZBTB7A_(SC-34508) |
| wgEncodeRegTfbsClusteredV2 | Combined | ZEB1_(SC-25388) |
| wgEncodeRegTfbsClusteredV2 | Combined | Znf143_(16618-1-AP) |
| wgEncodeRegTfbsClusteredV2 | Combined | ZNF263 |
| wgEncodeRegTfbsClusteredV2 | Combined | ZNF274 |
| wgEncodeRegTfbsClusteredV2 | Combined | ZZZ3 |
