# Supplementary material for: Systematic classification of non-coding RNAs by epigenomic similarity
Source: BMC Bioinformatics. 2013 Oct 9;14(Suppl 14):S2. doi: 10.1186/1471-2105-14-S14-S2 (PMC3851203; doi:10.1186/1471-2105-14-S14-S2)
Supplement: Additional file 2 — Cell types used to obtain cell type-specific epigenomic elements. The vocabulary is extracted from the ENCODE Consortium Data Coordination Center at UCSC (http://genome.ucsc.edu/ENCODE/cellTypes.html). M/F/U indicate cell/tissue donor was of male, female, or unknown gender, respectively. [file 1471-2105-14-S14-S2-S2.docx]

| **Cell Line/Type** | **Description** | **Cell Lineage/ Tissue** | **Gender** | **Vendor ID** |
| --- | --- | --- | --- | --- |
| **GM12878** | B-lymphoblastoid, normal karyotype, European Caucasian, Epstein-Barr Virus | mesoderm/blood | F | Corriell GM12878 |
| **H1hESC** | Human Embryonic Stem Cells, normal karyotype | inner cell mass/embryonic stem cells | M | WiCell Research Institute WA01 |
| **HeLa-S3** | Cervical carcinoma, African American, clonal derivative of the HeLa line, adapted for use in spinner culture, human papilloma virus (HPV-18) | ectoderm/cervix | F | ATCC CCL-2.2 |
| **HepG2** | Hepatocellular carcinoma, Caucasian | endoderm/liver | M | ATCC HB-8065 |
| **HMEC** | Human Mammary Epithelial Cells, normal karyotype | ectoderm/mammary gland | F | Lonza CC-2551 |
| **HSMM** | Human Skeletal Muscle Myoblasts, normal karyotype | mesoderm/muscle | U | Lonza CC-2580 |
| **HSMMtube** | Normal Human Skeletal Myotubes differentiated from HSMM cells (Bernstein protocol; http://genome.ucsc.edu), normal karyotype | mesoderm/muscle | U | Lonza CC-2580 |
| **HUVEC** | Human Umbilical Vein Endothelial Cells, normal karyotype | mesoderm/blood vessel | U | Lonza CC-2517 |
| **K562** | Lymphoblast leukemia cell line from a chronic myelogenous leukemia patient in terminal blast crisis | mesoderm/blood | F | ATCC CCL-243 |
| **NH-A** | Normal Human Astrocytes, normal karyotype | ectoderm/brain | U | Lonza CC-2565 |
| **NHDF-Ad** | Normal human Adult Dermal Fibroblasts, normal karyotype | mesoderm/skin | U | Lonza CC-2511 |
| **NHEK** | Normal Human Epidermal Keratinocytes, normal karyotype | ectoderm/skin | U | Lonza CC-2501 |
| **NHLF** | Normal Human Lung Fibroblast, normal karyotype | endoderm/lung | U | Lonza CC-2512 |
| **Osteobl** | Normal Human Osteoblasts, normal karyotype | mesoderm/bone | U | Lonza CC-2538 |

**Supplemental Table 2.** Cell types used to obtain cell type-specific epigenomic elements. The vocabulary is extracted from the ENCODE Consortium Data Coordination Center at UCSC (<http://genome.ucsc.edu/ENCODE/cellTypes.html>). M/F/U indicate cell/tissue donor was of male, female, or unknown gender, respectively.
